# Resveratrol Suppresses Severe Acute Pancreatitis-Induced Microcirculation Disturbance through Targeting SIRT1-FOXO1 Axis

**DOI:** 10.1155/2021/8891544

**Published:** 2021-02-09

**Authors:** Yuping Rong, Jun Ren, Wei Song, Renshen Xiang, Yuhang Ge, Wei Lu, Tao Fu

**Affiliations:** ^1^Department of Pancreatic Surgery, Renmin Hospital of Wuhan University, No. 238, Jiefang Road, Wuhan, Hubei, China 430060; ^2^Department of Gastrointestinal Surgery II, Renmin Hospital of Wuhan University, No. 238, Jiefang Road, Wuhan, Hubei, China 430060

## Abstract

**Background:**

Resveratrol (RSV), one of the SIRT1 agonists, has the ability of alleviating severe acute pancreatitis (SAP); however, the concrete protective mechanism remains unknown. It is noteworthy that microcirculation disturbance plays a vital role in SAP, and the SIRT1/FOX1 axis can regulate microcirculation. Therefore, this study is aimed at ascertaining what is the underlying mechanism of the protective effect of RSV on SAP, and whether it is associated with alleviating microcirculation disturbance by regulating the SIRT1/FOX1 axis.

**Method:**

The model of SAP was induced by retrograde injection of sodium taurodeoxycholate into the bile duct of the rats. The pancreatic wet/dry weight, ET/NO, and TXB_2_/6-keto-PGF_1*α*_ ratios; microcirculatory function; and SIRT1 activity were examined. ELISA was used to examine the serum level of lipase, amylase, hemorheology, ET, NO, TXB_2_, and 6-keto-PGF_1*α*_ and the content of SIRT1, VEGF, Ang I, and Ang II in the pancreas. RT-PCR was used to examine the mRNA level of VEGF, Ang I, and Ang II. Western blotting was used to detect SIRT1, FOXO1, and acetyl-FOXO1. Immunoprecipitation was used to examine the interaction of SIRT1 and FOXO1.

**Results:**

Resveratrol can significantly decrease the expression of lipase, amylase, acetyl-FOXO1, VEGF, Ang II, ET, NO, TXB_2_, and 6-keto-PGF_1*α*_ and the ratio of wet/dry weight, ET/NO, and TXB_2_/6-keto-PGF_1*α*_ by improving microcirculatory dysfunction and blood viscosity in SAP. Moreover, resveratrol can also promote the interaction of SIRT1 and FOXO1 and increase SIRT1 activity and the expression of SIRT1 and Ang I. The SIRT1 inhibitor, Sirtinol (EX527), obliviously reversed the effects of RSV on SAP.

**Conclusion:**

Resveratrol can protect rats against SAP, and its protective mechanism is associated with suppressing microcirculation disturbance through activating SIRT1-FOXO1 axis.

## 1. Introduction

Acute pancreatitis (AP) is a common and frequently occurring disease in clinics, and 10%-20% patients will eventually develop into severe acute pancreatitis (SAP), which is one of the most dangerous acute abdominal conditions, with many complications and high mortality [1]. Moreover, the main cause of SAP includes hyperlipidemia, heavy drinking, and bile duct stone obstruction [1]. Although the level of its diagnosis and treatment has been improved in recent years, the overall mortality rate is still as high as 17-30% [2]. Therefore, developing a new strategy to alleviate SAP damage and improve clinical outcome is an urgent clinical problem to be solved. In view of this, it is particularly important to clarify the pathogenesis of SAP.

It is widely acknowledged that the occurrence of acute pancreatitis is closely related to pancreatic microcirculation disorder, which is involved in the complex pathophysiological process from acute pancreatitis to severe acute pancreatitis [3]. The decrease of local perfusion caused by pancreatic microcirculation dysfunction may be the main factor that promotes the necrosis and diffusion of pancreatic acinar cells [3]. Improving pancreatic microcirculation function can slow down the progression of the disease and reduce the mortality rate [3]. Therefore, it is an effective approach to improving SAP by cutting down the microcirculation dysfunction of the pancreas.

Silent information regulator 1 (SIRT1), one member of the Sirtuin family, belongs to nicotinamide adenine dinucleotide- (NAD^+^-) dependent histone deacetylase and widely exists in human tissues and cells [4]. A previous study demonstrated that SIRT1 not only regulates physiological activities through histone and nonhistone deacetylation but also regulates many transcription factors, including p53, FOXO, E2F1, and NF-*κ*B [5]. Especially, it is worth noting that forkhead transcription factor O1 (FOXO1), one member of the FOXO family can regulate microcirculation, hemodynamics, and vascular endothelial function [6–8]. Therefore, it may be a promising strategy to attenuate microcirculation dysfunction of the pancreas through improving the SIRT1-FOXO1 axis-mediated microcirculation, hemodynamics, and vascular endothelial function of the pancreas.

Resveratrol (RSV), a representative SIRT1 agonist, is mainly found in grape skins, mulberry, peanut, and other traditional Chinese medicines and foods. It has biological activities such as prevention of tumor, inhibition of inflammation, improvement of circulation, antiatherosclerotic activities in the elderly, and inhibition of cell proliferation [9]. Recent studies displayed that RSV can alleviate SAP by promoting SIRT1 activation [10]. However, the exact mechanism is still unclear and needs further study. Therefore, the aim of the study is to investigate the protective effect of RSV on SAP and its possible mechanisms.

## 2. Materials and Methods

### 2.1. Animals, Drugs, and Reagents

Eighty male SD rats (6-8 weeks old) weighing 180-220 g were purchased from the experimental animal center of Wuhan University. They were reared under natural light, with a constant temperature of 25°C, relative humidity of 50%, and noise levels less than 60 dB. During the period, the cages were cleaned regularly. The rats were free to drink water and eat with a regularly maintained clean feeding environment, ventilation for 8-12 times/h, ammonia concentration ≤ 20, and flow rate for 10-25 cm/s. The study was authorized and approved by the Animal Care and Use Committee of Renmin Hospital of Wuhan University. All research steps were strictly in accordance with the NIH guidelines for Laboratory Animals.

Resveratrol (RSV, purity > 99%, No. R5010), dimethyl sulfoxide (DMSO, No. 276855), and sirtinol (EX527, No. E7034) were purchased from Sigma-Aldrich. Pentobarbital for anesthesia was purchased from Shenzhen Reward Life Science and Technology Co., Ltd. Sodium taurocholate was purchased from Shanghai Dibao Biology Technology Co., Ltd. (No. 145-42-6). Antibodies for SIRT1 (No. 2310), FOXO1 (No. 14262), acetyl-FOXO1 (No. 9464), *β*-actin (No. 4967), and IgG secondary (No. 4418) were all obtained from Cell Signaling Technology. Kits for lipase (MA1-10611), amylase (MA1-34918), and SIRT1 (MA5-30879) were purchased from eBioscience (California, USA). Kits for endothelin (ET, H093), nitric oxide (No. A012-1-2), and thromboxane B2 (TXB_2_, H331) were purchased from Nanjing Jiancheng Technology Co., Ltd. 6-Keto-PGF_1*α*_ was purchased from R&D Systems Inc. (USA, Minnesota, SND-R714). Kits for vascular endothelial growth factor (VEGF, EK383/2-96), angiopoietin I (Ang I, EK1017), and angiopoietin II (Ang II, EK1021) were provided by Shanghai Baiye Biotechnology Center. Resveratrol and EX527 were dissolved in 5% DMSO.

### 2.2. Animal Grouping

80 SD rats were equally divided into 4 groups: the sham group (Sham), the severe acute pancreatitis group (SAP), the resveratrol treatment group (RSV), and the RSV+EX527 group (RSV+EX, EX) (*n* = 20). The rats in the RSV group were treated with RSV (30 mg/kg) by intraperitoneal injection at 12 hours after the establishment of the SAP model; the rats in the EX group were treated with RSV (30 mg/kg) and EX527 (5 mg/kg) by intraperitoneal injection at 12 hours after the establishment of the SAP model [11, 12]; the rats in the sham and SAP groups were given an equal volume of DMSO diluted with saline by intraperitoneal injection; the rats in the SAP, RSV, and EX groups were given a retrograde injection of sodium taurodeoxycholate (1 mL/kg) into the bile duct to induce SAP [11, 12]. The rats in the sham group underwent the same operation except for a retrograde injection of sodium taurodeoxycholate into the bile duct.

### 2.3. Establishment of SAP Model

The model of SAP is induced as follows. Rats were fasted for 12 hours and were allowed to drink for 4 hours before the operation. Then, the rats were anesthetized with 3% pentobarbital sodium (1 mL/kg) through intraperitoneal injection. After anesthesia was successful, the rats were fixed on the operating table and their abdominal hair was cut off. After skin disinfection, a median incision was performed on the abdomen layer by layer. The abdominal cavity was opened to find the duodenal papilla, which is the position where the common bile duct and pancreatic duct converge into the duodenum. The proximal common bile duct was clipped by a minimally invasive vascular clamp. One needle was used for suturing with a Prolene suture on the distal common bile duct, and the suture was loosened and fixed. The needle was inserted from the duodenal papilla to the proximal end of the common bile duct. After the fine needle entered the common bile duct, the distal end of the common bile duct was ligated. An automatic micropump was used to slowly inject 3% sodium taurocholate into the distal end of the common bile duct. When the pancreatic tissue was filled with sodium taurocholate mixed with methylene blue and was visible by naked eyes, this represents that the model of SAP was established successfully. The minimally invasive vascular clamp is loosened and followed by loosening the Prolene thread. The duodenal needle entry point is sutured, the instruments are counted, and the abdomen is closed layer by layer. The concrete experimental steps are based on reference [12]. After 3 days of normal feeding, all rats in each group were anesthetized with 3% pentobarbital sodium (1 mL/kg) and fixed on the operating table with a constant-temperature electric blanket (37 ± 0.5°C). The abdominal cavity is opened, and 2 tubes of blood samples are taken from the abdominal aorta; each tube has 5 mL. One of them is an EDTA anticoagulant tube, and the other tube is a common blood collection tube. Then, rats were euthanized by cervical dislocation and pancreatic tissues were collected.

### 2.4. Testing Hemorheology

An EDTA anticoagulant tube was taken, and erythrocyte aggregation index; erythrocyte rigidity index; and high, medium, and low shear viscosity of whole blood were measured by a rapid hemorheology analyzer.

### 2.5. Examining the Pancreatic Microcirculation Function

The rats in very group were treated by injecting FITC-RBC (1.5 mL) through the tail vein. The pancreas was free from the abdominal cavity and spread on the transparent window of the perfusion box filled with normal saline, and maintained stable for 5 min. The microscopic images were harvested by a fluorescence microscope. The microcirculation observation system software of the BI-2000 Medical Image Analysis System was used to analyze the red blood cell flow, flow velocity, blood vessel number, and functional blood vessel number.

### 2.6. Measuring Wet/Dry Weight Ratio of Pancreas

To obtain the wet weight, pancreatic tissue is taken from liquid nitrogen and weighed. To obtain the dry weight, the pancreatic tissue was dried at 60°C for 48 h and weighed. Finally, the wet/dry weight ratio of the pancreas was calculated.

### 2.7. Detection of Serum Indexes

The blood samples were separated by centrifugation after blood coagulation. The expression of amylase, lipase, ET, NO, TXB_2_, and 6-keto-PGF_1*α*_ in the serum was determined by radioimmunoassay. The concrete steps were based on the manufacturer's instructions.

### 2.8. Assaying VEGF, SIRT1, Ang I, and Ang II Protein in Pancreatic Homogenate

The concentrations of SIRT1, VEGF, Ang I, and Ang II in the pancreas homogenate were determined by ELISA kits for SIRT1, VEGF, Ang I, and Ang II. The concrete steps were based on the instructions that came along with the kits.

### 2.9. Detection of VEGF, Ang I, and Ang II mRNA Expression in Pancreas

The pancreatic tissue was ground with liquid nitrogen and total RNA was extracted by a TRIzol kit. After determining and adjusting the concentration and purity of total RNA, a reverse transcription kit was used to reverse transcribe total RNA into total cDNA. The cDNA fragment was amplified by PCR using specific primers (Takara Company, Dalian, China). The special primer sequence is shown in Table 1. PCR was initiated in a thermal cycle programmed at 95°C for 5 min, 30 cycles of 94°C for 1 min, 60°C for 50 s, and 72°C for 1 min, and followed by extension at 72°C for 10 min and heat preservation at 4°C for 20 min. An image analysis instrument was used for scanning and analyzing. *β*-Actin was used as a housekeeping gene. The relative expression levels of VEGF, Ang I, and Ang II mRNA were calculated by the 2^−ΔΔCT^ method.

### 2.10. Western Blotting for SIRT1, FOXO1, Acetyl-FOXO1 Expression, and the Interaction of SIRT1 and FOXO1

After grinding the pancreatic tissue with liquid nitrogen, the total protein was extracted by protein lysate; then, the concentration of total protein was determined and adjusted. The protein lysate and the sample buffer were mixed, and the protein was separated by twelve alkyl sulfate polyacrylamide gel electrophoresis (SDS-PAGE) and transferred to the nitrocellulose membrane. The protein was blocked with PBS containing 50 g/L skimmed milk powder for 1 h at room temperature of 22°C. After that, it was incubated with the first antibodies against SIRT1 (1 : 500), FOXO1 (1 : 1000), acetyl-FOXO1 (1 : 400), and *β*-actin (1 : 3000) at 4°C overnight. After 3 times of PBS flushing, the electroluminescent solution was applied for developing and the gel imaging system was used for exposure imaging. The optical density (OD) of each strip was determined by Gel Pro Analyzer 4.0 software. The relative expression levels of SIRT1, FOXO1, and acetyl-FOXO1 were calculated with *β*-actin as internal reference.

### 2.11. Immunoprecipitation

The total protein was extracted from the pancreas of rats. The samples were collected and dissolved in an immunoprecipitation lysis buffer containing 25 mmol/L Tris, 150 mmol/L NaCl, 1 mmol/L EDTA, 1% NP-40, 1% SDS, 0.5% Triton-X, and 5% glycerol (pH 7.4). Homogenate was incubated on ice for 15 min and centrifuged at 4°C for 15 min. The lysate was incubated with the first antibody (2 *μ*g IgG) and normal IgG (2 *μ*g, as control) at 4°C overnight. Subsequently, 20 *μ*L protein was added. The A/G agarose beads were added to the suspension of the antigen-antibody complex and incubated for 2 h at 4°C on a shaker. The immune complex was obtained by centrifugation at 3000 R/min at 4°C for 3 min and washed with immunoprecipitation lysis buffer 3 times. 12% SDS-PAGE and PVDF were used to transfer the membrane and block it for 1 h. After incubation with anti-SIRT1 and anti-FOXO1 primary antibodies for 1 h, overnight at 4°C, the samples were incubated with HRP-labeled goat anti-rabbit or goat anti-mouse secondary antibody (1 : 3000) for 1 h at room temperature. The ECL chemiluminescence method was used to emit light in the cassette.

### 2.12. SIRT1 Activity Assay

The activity of SIRT1 was evaluated by the use of SIRT1 Activity Fluorescent Assay *μ*Kit (Beyotime, China) based on the manufacturer's instructions. The emission and excitation wavelengths were set at 460 nm and 360 nm.

### 2.13. Statistical Methods

All data were processed by SPSS 20.0 statistical software. All the data in this experiment were in accordance with normal distribution and expressed as mean ± standard deviation. Analysis of variance was performed among multiple groups. The SNK-q test was used for pairwise comparison within the group. *P* < 0.05 indicated that the difference was significant.

## 3. Results

### 3.1. Effects of RSV on the Pancreatic Microcirculation in SAP

Impaired pancreatic microcirculation is an important mechanism leading to SAP [3]. Therefore, we examine the effect of RSV on red blood cell flow, flow velocity, blood vessel number, and functional blood vessel number. As shown in Table 2, the result displayed that compared with the sham group, the SAP group had significantly lower red blood cell flow, blood flow velocity, and the number of blood vessels and functional vessels (*P* < 0.05). Compared with the SAP group, the RBC flow, blood flow velocity, the number of blood vessels, and the number of functional blood vessels in the RSV group were significantly increased (*P* < 0.05). However, the effects of RSV on pancreatic microcirculation were reversed with EX intervention as displayed by lower red blood cell flow, flow velocity, blood vessel number, and functional blood vessel number in the EX group compared to the RSV group (*P* < 0.05).

### 3.2. Effects of RSV on the Wet/Dry Weight Ratio of the Pancreas and the Expression of Serum Amylase and Lipase in SAP

Compared with the sham group, the wet/dry weight ratio of the pancreas and the serum expression of amylase and amylase in the SAP group were significantly increased (*P* < 0.05). Compared with the SAP group, the RSV group had a lower wet/dry weight ratio of the pancreas and the serum expression of amylase and amylase which indicated that RSV can significantly decrease the SAP-induced increase on the wet/dry weight ratio of the pancreas and the serum expression of amylase and amylase (*P* < 0.05). However, EX intervention can offset the effect of RSV in SAP as shown by the higher wet/dry weight ratio of the pancreas and the serum expression of amylase and amylase in the EX group compared to the RSV group (*P* < 0.05) (Table 3);

### 3.3. Effects of RSV on the Hemorheology in SAP

A rapid hemorheological measuring instrument was used to test for hemorheological properties including erythrocyte aggregation index; erythrocyte rigidity index; and high, medium, and low shear viscosity of whole blood. As displayed in Table 4, compared with the sham group, the erythrocyte aggregation index; erythrocyte rigidity index; and whole blood high, medium, and low shear viscosity in the SAP group were significantly increased (*P* < 0.05). Compared with the SAP group, the erythrocyte aggregation index; erythrocyte rigidity index; and whole blood high, medium, and low shear viscosity in the RSV group were significantly decreased (*P* < 0.05). Moreover, the EX group had higher erythrocyte aggregation index; erythrocyte rigidity index; and whole blood high, medium, and low shear viscosity than the RSV group (*P* < 0.05).

### 3.4. Effects of RSV on the Angiogenesis in SAP

VEGF, Ang I, and Ang II play an important role in angiogenesis, maturation, remodeling, and stabilization [13, 14]. Therefore, we examine the mRNA and protein levels of VEGF, Ang I, and Ang II. As shown in Tables 5 and 6, compared with the sham group, the expression of VEGF and Ang II was significantly increased in the SAP group with a decrease in the expression of Ang I (*P* < 0.05). Compared with the SAP group, the expression of VEGF and Ang II in the RSV group was significantly decreased with an obviously increasing Ang I expression (*P* < 0.05). Compared with the RSV group, the expression of VEGF and Ang II was significantly increased in the EX group with a decrease in the expression of Ang I (*P* < 0.05).

### 3.5. Effects of RSV on the Vascular Endothelial Cell Function in SAP

As shown in Table 7, compared with the sham group, the expression of ET, NO, TXB_2_, and 6-keto-PGF_1*α*_ and the ratio of ET/NO and TXB_2_/6-keto-PGF_1*α*_ were significantly increased in the SAP group (*P* < 0.05). Compared with the SAP group, the expression of ET, NO, TXB_2_, and 6-keto-PGF_1*α*_ and the ratio of ET/NO and TXB_2_/6-keto-PGF_1*α*_ were significantly decreased in the RSV group (*P* < 0.05). Compared with the RSV group, the expression of ET, NO, TXB_2_, and 6-keto-PGF_1*α*_ and the ratio of ET/NO and TXB_2_/6-keto-PGF_1*α*_ were significantly increased in the EX group (*P* < 0.05).

### 3.6. Effects of RSV on the SIRT-FOXO1 Axis and the Interaction of SIRT and FOXO1 in SAP

**Figure 1:**
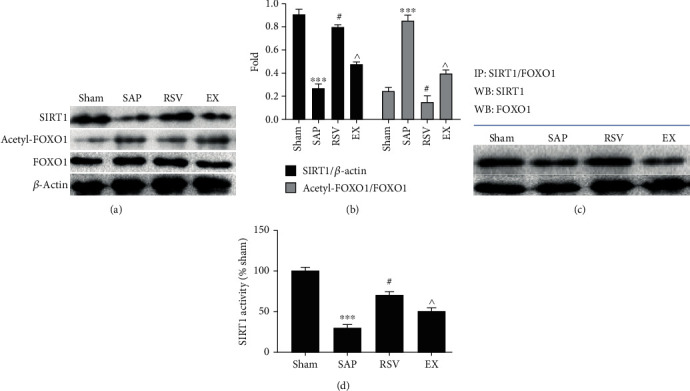
The effect of RSV on SIRT1 activation; FOXO1-SIRT1 interaction; and FOXO1, acetyl-FOXO1, and SIRT1 expression in SAP. Western blot analysis was employed on the expression of SIRT1, FOXO1, and acetyl-FOXO1. (a) Representative results for Western blot analysis of SIRT1, FOXO1, and acetyl-FOXO1 in the pancreases. (b) Semiquantitative analysis of 10 animals studied in each group. The relative amounts of SIRT1, FOXO1, and acetyl-FOXO1 in each group of rats were normalized by *β*-actin (a). Bars represent the means ± SE (*n* = 10). ^∗∗∗^*P* < 0.05 (SAP vs. sham); ^#^*P* < 0.05 (RSV vs. SAP); ^*P* < 0.05 (EX vs. RSV). (c) Immunoprecipitation was employed in the interaction of SIRT1 and FOXO1. (d) The activity of SIRT1.

The SIRT-FOXO1 axis plays an important role in regulating microcirculation [7, 8]. Therefore, we evaluated the activation of the SIRT-FOXO1 axis and the interaction of SIRT and FOXO1. As shown in Figure 1, the results show that compared with the sham group, the SIRT1 expression, activity, and the interaction of FOXO1 were significantly decreased in the SAP group with an obviously increasing acetyl-FOXO1 expression (*P* < 0.05). Compared with the SAP group, the SIRT1 expression, activity, and the interaction of FOXO1 were significantly increased in the RSV group with an obviously decreasing acetyl-FOXO1 expression (*P* < 0.05). Compared with the RSV group, the SIRT1 expression, activity, and the interaction of FOXO1 were significantly decreased in the EX group with an obviously increasing acetyl-FOXO1 expression (*P* < 0.05). Moreover, there is no difference on the expression of FOXO1 among the four groups (*P* > 0.05).

### 3.7. The Structure of RSV

**Figure 2:**
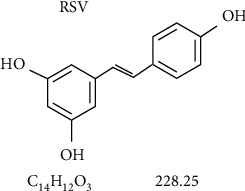
The structure of RSV.

The structure of RSV is shown in Figure 2.

## 4. Discussions

The pathogenesis and treatment of severe acute pancreatitis has been a difficult and hot topic in recent years [1]. With the deepening of the pathogenesis and clinical research of SAP, the treatment of SAP has attained important progress [1]. The mortality rate of SAP is significantly lower than before [1, 2]. The vast majority of patients with SAP have achieved a satisfactory curative effect through nonsurgical treatment [1, 2].

Many studies have suggested that the occurrence of pancreatic microcirculation dysfunction is related to its own blood supply characteristics, and the expression of amylase, lipase, and the ratio of the wet/dry weight of pancreas are typical markers of pancreatic injury [3, 10]. The pancreas is supplied with blood by a single branch of the intralobular artery, which is the anatomical basis of pancreatic microcirculation highly sensitive to ischemia. Moreover, the disturbance of the pancreatic microcirculation function is an important mechanism leading to SAP [3, 10]. The results showed that resveratrol can significantly increase the red blood cell flow, blood flow velocity, vascular number, and functional vascular number of pancreatic microvessels in SAP rats and reduce the expression of serum amylase and lipase, and the ratio of the wet/dry weight of pancreas. Moreover, EX527, a SIRT1 inhibitor, effectively abated the effect of RSV on pancreatic microcirculation and markers of pancreatic injury in SAP. This indicated that RSV can significantly alleviate SAP by improving pancreatic microcirculation.

VEGF is a glycoprotein, which can promote the formation of new blood vessels and improve blood vessel permeability [13]. Moreover, it is the strongest microvascular high-permeability inducer known so far. VEGF can increase the migration of endothelial cells and the formation of new blood vessels, and it can promote the secretion of matrix metalloproteinases from endothelial cells and their surrounding cells under the regulation of various factors which cause vascular fluid and protein extravasation into the tissues, then resulting in the decrease of tissue perfusion [13]. Ang I and Ang II/TIE2 are a new angiogenesis signal transduction pathway discovered after VEGF and plays an important role in angiogenesis, maturation, remodeling, and stabilization through participation in physiological and pathological angiogenesis under the synergism with VEGF [14]. Ang I may play a role in stabilizing the vascular network by targeting adhesion molecules to cell junctions and strengthening cell connections [14]. Ang II is an antagonist of Ang I. In the presence of angiogenic factors such as VEGF, Ang II can degenerate blood vessels and reduce the number of blood vessels [14]. Moreover, Ang II can also promote the apoptosis of endothelial cells and regression of blood vessels by inhibiting the vascular stability of Ang I [14]. The results showed that RSV can significantly decrease the expression of VEGF and Ang II with a decrease in Ang I expression. EX527 can reverse the effect of RSV on VEGF, Ang I, and Ang II which suggests that RSV can improve pancreatic microcirculation by promoting angiogenesis, maturation, remodeling, and stabilization.

Blood viscosity is one of the indexes reflecting hemodynamics [15]. The increase of blood viscosity leads to microcirculation disturbance [15]. The erythrocyte aggregation index and erythrocyte rigidity index are closely related to blood viscosity [15]. The results showed that RSV could significantly reduce the cell aggregation index, erythrocyte rigidity index, whole blood shear viscosity, and blood viscosity of SAP rats. EX can abolish the effect of RSV on blood viscosity in SAP. This shows that RSV can improve blood viscosity in SAP. SAP secretes a lot of fluid from the pancreas and its surrounding areas. The body is in a state of severe stress reaction [15]. Microvascular endothelial cells of the pancreas and other organs are damaged, leading to a release of cytokines and vasoactive mediators [15]. Vasoactive mediators include a vasoconstrictor and a vasodilator. Vasodilator substances mainly include ET and TXB_2_, while vasodilator substances mainly include NO and PGI_2_ [15]. In addition, ET can reduce pancreatic blood flow by inducing pancreatic microvascular spasm and overload calcium in acinar cells, damage pancreatic cells, and cause acinar necrosis [15]. The dynamic balance of NO can reflect the functional state of microcirculation [16]. TXB_2_ and PGI_2_ also play an important role in microcirculation ischemia. TXA_2_ can strongly constrict blood vessels and gather platelets [16]; PGI_2_ is a vasodilator, which can strongly dilate blood vessels and inhibit platelet aggregation and activation [16]. Therefore, TXB_2_/PGI_2_ are a pair of vasotonic mediators, and their imbalance leads to a dysfunction of pancreatic vascular movement. TXB_2_ and PGI_2_ are extremely unstable and are rapidly degraded into stable metabolites TXB_2_ and 6-keto-PGF_1*α*_, respectively [16]. The results show that RSV can significantly decrease the expression of ET, NO, TXB_2_, and 6-keto-PGF_1*α*_ and the ratio of ET/NO and TXB_2_/6-keto-PGF_1*α*_ in SAP rats. However, EX527 can attenuate the effect of RSV on the above index which indicates that RSV could significantly improve pancreatic microcirculation by improving the imbalance of ET/NO and TXB_2_/6-keto-PGF_1*α*_ in SAP.

The SIRT protein family is a highly conserved protein family in mammals. SIRT1 is the first SIRT protein discovered and is also the most studied SIRT protein [4]. SIRT1 is a NAD^+^-dependent histone deacetylase involved in many biological processes, and it can deacetylate the activities of various transcription factors and nonhistone substrates, including FOXO1 and ac-p53 [5]. FOXO1 is a member of the O-box subfamily. FOXO1 is regulated by acetylation, phosphorylation, and ubiquitination [6–8]. A previous study confirmed that active FOXO1 exists in the nucleus, and SIRT1 promotes FOXO1 transfer into the cytoplasm by deacetylation [6–8]. The SIRT1 promoter region contains a forkhead-like binding site, which can interact with FOXO1 then decrease acetylation of FOXO1-mediated signaling pathways [6], such as microcirculation, hemodynamics, and vascular endothelial function [10]. This result shows that RSV can increase the SIRT1 expression and activity and the interaction of FOXO1 with an obviously decreasing acetyl-FOXO1 expression. However, the SIRT1 inhibitor can reverse the effect of RSV on the SIRT-FOXO1 axis in SAP. This implies that the SIRT1 agonist can improve microcirculation, hemodynamics, and vascular endothelial function by regulating the SIRT-FOXO1 signal pathway.

In conclusion, resveratrol can significantly improve the pancreatic microcirculation of SAP rats, inhibit the expression of VEGF and Ang II, promote the expression of Ang I, reduce blood viscosity, and correct the disorder of ET/NO and TXA_2_/6-keto-PGF_1*α*_, which has a certain therapeutic effect on SAP rats and the mechanism is associated with targeting the SIRT-FOXO1 axis.

## Figures and Tables

**Table 1:** The primer for RT-PCR.

Gene	Sense (5′-3′)	Antisense (5′-3′)
VEGF	CAACTTCTGGGCTCTTCTCG	CCTCTCCTCTTCCTTCTCTTCC
Ang I	GGAACCGAGCCTACTCACAG	GCATCCTTCGTGCTGAAATC
Ang II	ATGAAGGAGCAGAAGGACGA	GAAGGAGCGAGTTGTTGACC
*β*-Actin	CCCATCTATGAGGGTTACGC	TTTAATGTCACGCACGATTTC

**Table 2:** The effect of RSV on microcirculatory function of pancreas in SAP ($\bar{x}\pm s$, *n* = 20).

Group	Red blood cell flow number (/min)	Blood flow velocity (*μ*m/s)	Blood vessel number	Functional vessel number
Sham	85.32 ± 6.25	226.34 ± 13.28	11.26 ± 0.84	11.24 ± 0.97
SAP	18.36 ± 2.45^∗∗∗^	65.39 ± 5.40^∗∗∗^	5.35 ± 0.47^∗∗∗^	4.36 ± 0.45^∗∗∗^
RSV	56.49 ± 5.68^#^	136.17 ± 9.26^#^	9.27 ± 0.73^#^	8.79 ± 0.75^#^
EX	27.23 ± 3.7^^^	72.63 ± 6.5^^^	6.14 ± 0.58^^^	6.52 ± 0.71^^^

*Note*. The SAP group compared with the sham group, ^∗∗∗^*P* < 0.01; the RSV group compared with the SAP group, ^#^*P* < 0.05; the EX group compared with the RSV group, ^#^*P* < 0.05.

**Table 3:** The effect of RSV on pancreatic weight indexes, serum amylase, and lipase activity in SAP ($\bar{x}\pm s$, *n* = 20).

Group	Wet/dry weight ratio (×10^−3^)	Lipase (U/mL)	Amylase (U/mL)
Sham	2.8 ± 1.2	27.8 ± 6.5	105.2 ± 13.4
SAP	8.6 ± 1.9^∗∗∗^	386.5 ± 18.2^∗∗∗^	1465.1 ± 35.2^∗∗∗^
RSV	3.6 ± 1.3^#^	68.3 ± 7.2^#^	359.6 ± 19.6^#^
EX	6.9 ± 1.7^^^	326.9 ± 16.3^^^	1273.8 ± 29.7^^^

*Note*. The SAP group compared with the sham group, ^∗∗∗^*P* < 0.01; the RSV group compared with the SAP group, ^#^*P* < 0.05; the EX group compared with the RSV group, ^#^*P* < 0.05.

**Table 4:** Effect of RSV on the hemorheology in SAP ($\bar{x}\pm s$, *n* = 20).

Group	Erythrocyte aggregation index	Erythrocyte rigidity index	High shear viscosity (mPa·s)	Medium shear viscosity (mPa·s)	Low shear viscosity (mPa·s)
Sham	2.14 ± 0.12	3.11 ± 0.17	4.51 ± 0.52	5.62 ± 0.53	10.15 ± 0.83
SAP	2.66 ± 0.18^∗^	4.63 ± 0.22^∗^	7.76 ± 0.69^∗^	9.76 ± 0.84^∗^	17.24 ± 1.32^∗^
RSV	2.21 ± 0.13^#^	3.39 ± 0.18^#^	5.13 ± 0.57^#^	6.24 ± 0.56^#^	12.58 ± 0.87^#^
EX	2.53 ± 0.16^^^	4.35 ± 0.29^^^	7.31 ± 0.65^^^	8.35 ± 0.72^^^	16.72 ± 1.15^^^

*Note*. The SAP group compared with the sham group, ^∗∗∗^*P* < 0.01; the RSV group compared with the SAP group, ^#^*P* < 0.05; the EX group compared with the RSV group, ^#^*P* < 0.05.

**Table 5:** Effect of RSV on the expression of VEGF, Ang I, and Ang II in SAP ($\bar{x}\pm s$, *n* = 20).

Group	VEGF (per gm tissue)	Ang I (per gm tissue)	Ang II (per gm tissue)
Sham	110 ± 5	1200 ± 150	840 ± 90
SAP	566 ± 55^∗^	550 ± 60^∗^	1600 ± 170^∗^
RSV	225 ± 15^#^	920 ± 120^#^	1150 ± 120^#^
EX	478 ± 35^^^	645 ± 75^^^	1480 ± 160^^^

*Note*. The SAP group compared with the sham group, ^∗∗∗^*P* < 0.01; the RSV group compared with the SAP group, ^#^*P* < 0.05; the EX group compared with the RSV group, ^#^*P* < 0.05.

**Table 6:** Effect of RSV on the mRNA level of VEGF, Ang I, and Ang II in SAP ($\bar{x}\pm s$, *n* = 20).

Group	VEGF	Ang I	Ang II
Sham	1.03 ± 0.22	15.33 ± 6.12	32.51 ± 5.09
SAP	1.78 ± 0.41^∗^	9.06 ± 3.17^∗^	56.84 ± 8.73^∗^
RSV	1.15 ± 0.27^#^	12.44 ± 4.47^#^	41.25 ± 6.12^#^
EX	1.36 ± 0.33^^^	10.89 ± 3.56^^^	49.93 ± 7.11^^^

*Note*. The SAP group compared with the sham group, ^∗∗∗^*P* < 0.01; the RSV group compared with the SAP group, ^#^*P* < 0.05; the EX group compared with the RSV group, ^#^*P* < 0.05.

**Table 7:** The effect of RSV on the vascular endothelium function in SAP ($\bar{x}\pm s$, *n* = 20).

Group	ET (pg/mL)	NO (pg/mL)	ET/NO	TXB_2_ (pg/mL)	6-Keto-PGF_1*α*_ (pg/mL)	TXB_2_/6-keto-PGF_1*α*_
Sham	46.24 ± 5.65	156.26 ± 17.53	0.29 ± 0.04	248.27 ± 14.32	52.43 ± 6.26	4.75 ± 0.44
SAP	238.41 ± 15.72^∗∗∗^	468.11 ± 51.32^∗∗∗^	0.50 ± 0.06^∗∗∗^	1466.15 ± 61.59^∗∗∗^	138.57 ± 16.25^∗∗∗^	10.62 ± 0.92^∗∗∗^
RSV	68.34 ± 6.95^#^	268.72 ± 25.36^#^	0.25 ± 0.03^#^	578.92 ± 32.35^#^	68.87 ± 7.82^#^	8.53 ± 0.66^#^
EX	225.47 ± 13.26^^^	378.47 ± 44.26^^^	0.58 ± 0.07^^^	1398.76 ± 55.14^^^	124.12 ± 14.67^^^	11.27 ± 1.12^#^

*Note*. The SAP group compared with the sham group, ^∗∗∗^*P* < 0.01; the RSV group compared with the SAP group, ^#^*P* < 0.05; the EX group compared with the RSV group, ^#^*P* < 0.05.

## Data Availability

The data that support the findings of this study are available from the corresponding author upon reasonable request.

## References

[1] Yu J., Xu S., Wang W. X. (2012). Changes of inflammation and apoptosis in adrenal gland after experimental injury in rats with acute necrotizing pancreatitis. *Inflammation*.

[2] Mitchell R. M. S., Byrne M. F., Baillie J. (2003). Pancreatitis. *Lancet*.

[3] Kinnala P. J., Kuttila K. T., Grönroos J. M., Havia T. V., Nevalainen T. J., Niinikoski J. H. (2001). Pancreatic tissue perfusion in experimental acute pancreatitis. *The European Journal of Surgery*.

[4] Maiese K., Chong Z. Z., Shang Y. C., Wang S. (2011). Translating cell survival and cell longevity into treatment strategies with SIRT1. *Romanian Journal of Morphology and Embryology*.

[5] Lim J. H., Lee Y. M., Chun Y. S., Chen J., Kim J. E., Park J. W. (2010). Sirtuin 1 modulates cellular responses to hypoxia by deacetylating hypoxia-inducible factor 1*α*. *Molecular Cell*.

[6] Ponugoti B., Dong G., Graves D. T. (2012). Role of Forkhead Transcription Factors in Diabetes-Induced Oxidative Stress. *Experimental Diabetes Research*.

[7] Tanaka J., Qiang L., Banks A. S. (2009). Foxo1 links hyperglycemia to LDL oxidation and endothelial nitric oxide synthase dysfunction in vascular endothelial cells. *Diabetes*.

[8] Goettsch W., Gryczka C., Korff T. (2008). Flow-dependent regulation of angiopoietin-2. *Journal of Cellular Physiology*.

[9] Diaz-Gerevini G. T., Repossi G., Dain A., Tarres M. C., Das U. N., Eynard A. R. (2016). Beneficial action of resveratrol: how and why?. *Nutrition*.

[10] Wang N., Zhang F., Yang L. (2017). Resveratrol protects against L-arginine-induced acute necrotizing pancreatitis in mice by enhancing SIRT1-mediated deacetylation of p53 and heat shock factor 1. *International Journal of Molecular Medicine*.

[11] Liu D.-L., Song G.-D., Ma Z.-L., Song Z.-S. (2020). Efficacy of resveratrol in treating rat models with severe acute pancreatitis. *Jiangsu Medical Journal*.

[12] Xu S., Gao Y., Zhang Q. (2016). SIRT1/3 activation by resveratrol attenuates acute kidney injury in a septic rat model. *Oxidative Medicine and Cellular Longevity*.

[13] Greenberg J. I., Shields D. J., Barillas S. G. (2008). A role for VEGF as a negative regulator of pericyte function and vessel maturation. *Nature*.

[14] Rennel E. S., Regula J. T., Harper S. J., Thomas M., Klein C., Bates D. O. (2011). A human neutralizing antibody specific to Ang-2 inhibits ocular angiogenesis. *Microcirculation*.

[15] Plusczyk T., Bersal B., Menger M. D., Feifel G. (2001). Differential effects of ET-1, ET-2, and ET-3 on pancreatic microcirculation, tissue integrity, and inflammation. *Digestive Diseases and Sciences*.

[16] Lempinen M., Stenman U. H., Halttunen J., Puolakkainen P., Haapiainen R., Kemppainen E. (2005). Early sequential changes in serum markers of acute pancreatitis induced by endoscopic retrograde cholangiopancreatography. *Pancreatology*.

